# Role of the Polycystins in Cell Migration, Polarity, and Tissue Morphogenesis

**DOI:** 10.3390/cells4040687

**Published:** 2015-10-30

**Authors:** Elisa Agnese Nigro, Maddalena Castelli, Alessandra Boletta

**Affiliations:** Division of Genetics and Cell Biology, Dibit, IRCCS-San Raffaele Scientific Institute, Via Olgettina 58, 20132 Milano, Italy; E-Mails: nigro.elisa@hsr.it (E.A.N.); castelli.maddalena@hsr.it (M.C.)

**Keywords:** polycystin, polycystic kidney disease, epithelial morphogenesis, cell migration, cell polarity, cilia, renal cyst, planar cell polarity

## Abstract

Cystic kidney diseases (CKD) is a class of disorders characterized by ciliary dysfunction and, therefore, belonging to the ciliopathies. The prototype CKD is autosomal dominant polycystic kidney disease (ADPKD), whose mutated genes encode for two membrane-bound proteins, polycystin-1 (PC-1) and polycystin-2 (PC-2), of unknown function. Recent studies on CKD-associated genes identified new mechanisms of morphogenesis that are central for establishment and maintenance of proper renal tubular diameter. During embryonic development in the mouse and lower vertebrates a convergent-extension (CE)-like mechanism based on planar cell polarity (PCP) and cellular intercalation is involved in “sculpting” the tubules into a narrow and elongated shape. Once the appropriate diameter is established, further elongation occurs through oriented cell division (OCD). The polycystins (PCs) regulate some of these essential processes. In this review we summarize recent work on the role of PCs in regulating cell migration, the cytoskeleton, and front-rear polarity. These important properties are essential for proper morphogenesis of the renal tubules and the lymphatic vessels. We highlight here several open questions and controversies. Finally, we try to outline some of the next steps required to study these processes and their relevance in physiological and pathological conditions.

## 1. Introduction

Cystic kidney diseases (CKD) are a group of inherited pathologies characterized by dilatation or ballooning of the epithelia lining the renal tubule. As a whole, they represent the majority of all genetic disorders affecting the kidney. Polycystic kidney disease (PKD) is the prototype of these disorders that share several features at the molecular and cellular level [1,2,3,4].

The genes mutated in the various diseases encode for a very wide variety of proteins with distinct functional roles, whose activity converges on the fine regulation of the structure and/or function of a unique type of organelle, called the primary cilium. This is a single, microtubule-based structure protruding from most cells of our organism [4]. In epithelial and endothelial cells, cilia protrude from the apical side into the lumen of tubules or vessels. While the central role of cilia in renal cystic disorders is not of question, what the precise function of this organelle and how it can regulate the homeostasis of the renal tubule in physiological, as well as in pathological, conditions is far from being clear. For excellent reviews of the literature related to all cystic kidney diseases, the ciliopathies, and the structure/function of primary cilia we refer elsewhere [1,2,3,4].

In this review we will focus our attention on some recent work that has highlighted a potential key role of cell migration and planar cell polarity in the regulation of the normal homeostasis of the renal tubule, whose derangement is likely involved in the degeneration of normal renal epithelia into cysts. The central thread of this report will be recent findings demonstrating a central role of the polycystins (PCs), mutated in autosomal dominant polycystic kidney disease (ADPKD) in regulation of cell migration and polarity [5,6,7,8,9].

## 2. Planar Cell Polarity (PCP) in Tissue Morphogenesis: Lessons from Lower Organisms

**Figure 1:**
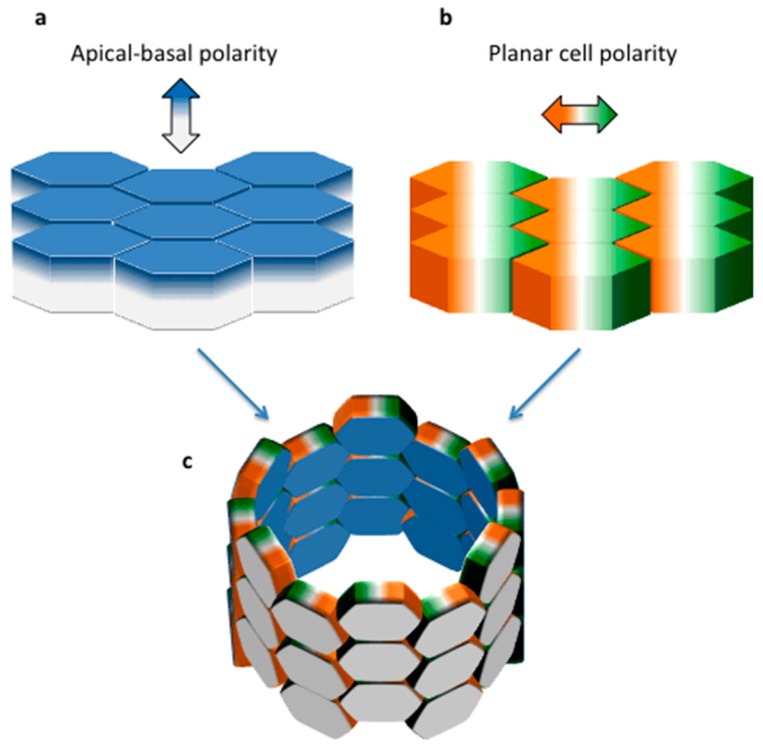
Apical-basal and Planar Polarity in Epithelia. Schematic representation of an epithelium, showing an apical-basal polarity along the vertical axis (**a**) and a planar cell polarity along the orthogonal axis (**b**); their appearance in association is shown in a tubular structure (**c**).

Epithelial cells are characterized by polarization along two orthogonal axes. Apical/basal polarity defines the orientation of the cells along the vertical axis relative to the matrix (localized below the base of the cell, Figure 1a). PCP is defined as the organization of cells within the plane of the tissue that is perpendicular to the apical/basal axis (Figure 1b,c) [10]. PCP is probably present in every existing epithelium, but it is best recognized and easier to study in those tissues in which each epithelial cell achieves an asymmetrical morphology that facilitates its visualization. In most epithelia in higher organisms, however, this does not occur. For this reason PCP has been extensively studied in lower organisms such as the fruit fly *Drosophila melanogaster* which provides many examples of PCP: in the wing hairs, body bristles and the eye [10]. The common principles are conserved across tissues and species [10,11]. The local alignment of cell polarity in the wing is guided by the so-called “core PCP pathway” and by the Fat/Dachsous (Ft/Ds) system (reviewed in [11]). Briefly, the core PCP pathway is composed of the multipass transmembrane proteins Frizzled (Fz), Van Gogh/Strabismus (Vang/Stbm), the atypical cadherin Flamingo/Starry night (Fmi/Stan), and of the cytoplasmic proteins Disheveled (Dsh), Diego, and Prickle (Pk). The Ft/Ds system includes the protocadherins Fat (Ft) and Dachsous (Ds) and the Golgi resident transmembrane kinase, Four-Jointed (Fj). Although the most evident examples of planar polarity are found in plain epithelia, like the wing of *Drosophila*, examples of planar polarity are also found in vertebrates. One of the best systems to study the core PCP signaling in mammals is the cochlear epithelium of the inner ear, showing a population of precisely-organized sensitive mechanosensory hair cells (reviewed in [12,13]).

PCP has been shown to direct several morphogenetic events, including those relying on convergent extension (CE) and oriented cell division (OCD) in a variety of organisms [11]. CE is a highly-regulated process that, by driving the change in the position of the cells in an epithelial monolayer, leads to tissues narrowing along one axis and concomitant extension along another axis. The first identified example of CE in development is body axis elongation during gastrulation (reviewed in [14]). During body axis elongation, germ layer progenitor cells move towards the dorsal side of the gastrula, where the embryonic body axis will form. Subsequently, cells intercalate along the axis of movement. This combination of collective cell movement and cell intercalations lead to the narrowing of the body axis along its medial-lateral axis (*i.e.*, the convergence) and elongation along its anterior-posterior axis (*i.e.*, the extension) [15]. CE includes two different types of cell movement: in collective migration, cells migrate as a cohesive sheet and do not intercalate; during medial-lateral cell intercalation, cells redistribute their positions in the anterior-posterior axis of the tissue [15]. In *Drosophila*, Par3, also known as Bazooka, mediates both of these mechanisms [16].

PCP pathway components are critical for cell intercalations and apical constriction during neural tube closure and mesoderm convergent extension [17,18,19,20,21]. Convergent extension is driven largely by mediolateral cell intercalations [22,23], PCP components were proposed to stabilize mediolateral cell protrusions [24,25] and/or promote actomyosin contractility at mediolaterally-oriented cell junctions [26].

The role of PCP in directing CE is demonstrated by imaging studies and by the presence of defective CE processes following disruption of core PCP complex components. All the major components of the Wnt/PCP pathway, such as Disheveled, Prickle, Fmi, Strabismus, Wnt5a, and Wnt11 have been shown to play a crucial role in directing CE movements during gastrulation and neurulation both in *Xenopus* and in zebrafish [24,25,27,28,29,30,31]. Of note, the roles of Wnt5a and Wnt11 have also been recently uncovered for anterior-posterior axis elongation in mammals [32].

As for CE, the PCP pathway also regulates OCD. OCD is determined by the position of the mitotic spindle and during animal development guides the correct elongation and shaping of tissues (reviewed in [33,34]). OCD has been extensively studied in zebrafish where, both in the dorsal and in the ventral region of the gastrula, cell divisions are highly-oriented along the animal-vegetal axis [35,36,37]. Similarly, in the *Xenopus* embryo, cells were shown to divide following three directions in relation to the embryonic surface: parallel, oblique, and perpendicular [38]. OCD is not an exclusive process of vertebrates, but it is also observed in lower organisms. During *Drosophila Melanogaster* embryogenesis the germ band extends and elongates in a process in which cells divide preferentially along the anterior-posterior axis, corresponding to the long axis of the extending tissue [39].

## 3. Establishment and Maintenance of Tubular Diameter in the Developing Kidney

**Figure 2:**
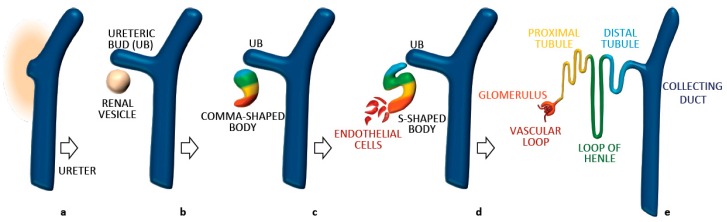
Main developmental steps of the renal nephron. Branching of the ureter generates ureteric buds (UB), surrounded by mesenchymal tissue (**a**); their interaction induces a mesenchymal-to-epithelial transition and generates the renal vescicle (**b**); that develops in a patterned comma-shaped body (**c**); subsequently extending in a tubular S-shaped body (**d**). On one side the S-shaped body makes contact with the ureter, on the other side it connects with migrating endothelial cells that will form the vascular loop within the glomerulus. The patterned expression of specific transcription factors in each tract of the S-shaped body generates the different structures of the mature nephron (see color code (**e**)).

**Figure 3:**
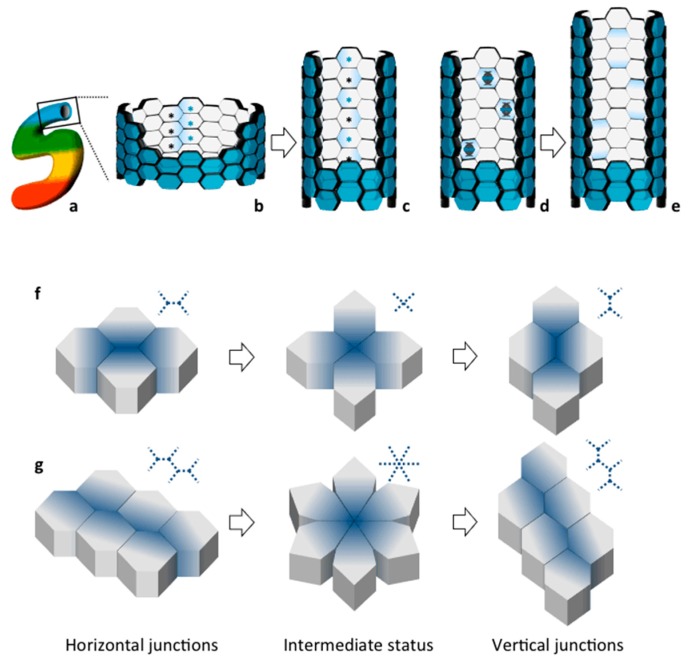
Narrowing and elongation of developing renal tubules and collecting ducts. During embryonic development, the S-shaped body tubular structure (**a**) elongates and establish its diameter in a proliferation-independent manner: because cells intercalate perpendicularly to the longitudinal axis, the tubule becomes narrower and longer without changing cell number (**b**,**c**); in post-natal phases, the longitudinal orientation of cell division allows to maintain the diameter and to increase length of the tubule (**d**,**e**). During cell intercalation, cells resolve from a horizontal status to a vertical one passing through an intermediate cross-shaped state (**f**) When re-shaping involves a larger number of cells, the intermediate status displays a rosette profile (**g**).

All processes described above, including CE and OCD, have been shown to take place during normal renal development and to contribute to tubular morphogenesis and elongation [40,41,42]. Each mammalian kidney is made of more than a million nephrons. Each nephron is made of a glomerulus and a tubule connecting to the collecting duct system (Figure 2). Fine shaping of the renal tubule is key for its proper function. This morphogenetic process takes place during development, which occurs both at the embryonic and at the neonatal stage in the mouse. During embryonic development, an epithelial structure called the ureteric bud (UB) invades a metanephric mesenchyme (MM) [40]. The first will undergo a series of branching events. The MM surrounding each UB tip undergoes a mesenchymal-to-epithelial transition to form comma and S-shaped bodies [40] (Figure 2). These are composed of an immature epithelium with a central lumen and an apical-basal axis already established [40,43]. Comma and S-shaped bodies next undergo a program of patterning and elongation necessary to generate the mature nephron [43]. Elongation occurs both in the tubules and the collecting ducts. But how and when is the final diameter of the renal tubule established? As it is often the case, human disorders have helped understanding the importance of specific biological processes. In this case, studies on CKDs that are characterized by defective establishment and/or maintenance of proper tubular diameter has paved the way to fundamental discoveries. Recent work has shown that establishment of tubular diameter occurs through two distinct processes in the mouse: during embryonic development elongation involves a process of CE movements driving cellular intercalation [5,42,44] (Figure 3a–e); once the correct tubular diameter has been established the elongation continues post-natally through OCD [41]. Importantly, interfering with either process has been associated to cyst formation [5,41,42,44], although the precise role for CE and OCD in cystogenesis remains unclear (see below). As described above, both of these processes rely on establishment of PCP.

We know very little about the molecular and the cellular determinants of these processes in the kidney. However, one could infer that the basic mechanism might be well conserved and the lower organisms might provide important information. Two types of movement have been proposed for cell intercalation. By shrinking the horizontal junction, cells converge to a central vertex leading to an intermediate state in which all cells interact by cross-shaped junctions and that, eventually, is extended in a vertical junction (Figure 3f). When re-shaping involves a larger number of cells, the shrinking of horizontal junctions results in formation of a rosette-shaped intermediate that might resolve perpendicularly in vertical junctions (Figure 3g). Of note, convergence of cells and subsequent perpendicular extension determine re-shape of tissue without changing cell number.

A major limitation preventing further understanding of the process stands in our current inability to visualize the process in living conditions in mammals. However, imaging of the process in living conditions was recently achieved in *Xenopus* [44] which is relatively easy to image because it is transparent and it contains a single nephron. The results confirmed active cell movement and cell-shape changes and described a novel rosette-based CE movement similar to the one shown in Figure 3g, providing an initial clue of how renal epithelia take their tubular shape, at least in the frog. This process also depends on PCP [44] as for all other examples of CE. However, we do not know how well conserved the process is in higher vertebrates. Static imaging of fixed kidneys confirmed the presence of rosette-like structures in the developing kidney in the mouse, suggesting that the mechanism might be well conserved [44]. According to this model, kidney cells become organized in rosettes and then elongate mediolaterally and move directionally to intercalate with neighboring cells (Figure 3g, [44]). Subsequently to the achievement of the optimal tubular diameter, further elongation of the tubule is reached by OCD [41,42], ensuring elongation, while preserving a correct diameter [44]. At the molecular level, very little is known about which molecules or pathways drive the process: Wnt9b via the non-canonical pathway, dishevelled-2, polycystin-1 (PC-1), and Par3 have so far been involved [5,42,44]. Of note, PC-1 was shown for the first time to be essential for the process of convergent extension in the developing murine kidney via its interaction and regulation of the Par3 polarity molecule [5] (see below).

## 4. The Polycystins: A Multitasking Complex

Polycystin-1 (PC-1) and polycystin-2 (PC-2) are two membrane proteins encoded by the *PKD1* and *PKD2* genes, mutated in ADPKD [9,45,46]. PC-1 is a large (~520 kDa) receptor with a long extracellular domain, 11 transmembrane domains, and a short intracellular C-tail of 198 aa, mediating signaling [9,45,46]. PC-2 is a Ca^++^ channel of the Transient Receptor Potential (TRP) channel family. The two proteins interact through a coiled-coil domain in their intracellular C-termini to form a complex [47]. Their association in a complex explains why ADPKD type I and type II are so similar. The complex is localized in cell-cell/matrix interacting structures, in extracellular components, such as exosomes, as well as at the primary cilium [9,45,46,48]. This is a microtubule-based solitary organelle present on the apical side of renal epithelia protruding into the tubular lumen [3,4]. Extensive evidence showed the central role of cilia in preventing renal cyst formation. In line with the fact that the PC-1/PC-2 complex plays a role in the regulation of calcium, cells derived from tissues of ADPKD patients show reduced levels of intracellular calcium [49]. Whether this function of the PC-1/2 complex is related to their activity at the primary cilium remains unclear. While cells lacking the *Pkd1* and *Pkd2* genes were reported to have a defective response to ciliary bending in the intracellular calcium levels [50], recent work has questioned the key role of the PCs in regulation of calcium entry into the primary cilium [51]. Furthermore, the central role of cilia in regulation of cytosolic calcium levels has also been largely put into question [52]. Thus, one central issue that remains unresolved is what the role of calcium regulation by the PCs and by cilia at large is. If the main activity of the PCs is indeed to regulate calcium homeostasis, than one possibility is that this function might not be driven from cilia [51,52].

Interestingly, it has been debated for a long time whether the PC-1–PC-2 interaction is a prerequisite for localization of the complex on the ciliary membrane. Some studies indicated that PC-2 requires PC-1 to localize to the cilium [39,50,53] while others showed that PC-2 can reach the surface independently of PC-1 [54]. In support of the last model is the finding that each protein has its own ciliary targeting signal [54,55] and that they can reach the membrane surface through different ways: PC-1 via the *trans*-Golgi network (TGN) [55] while PC-2 directly via the *cis*-Golgi compartment [56]. However, recent studies performed mainly on the endogenous proteins, demonstrated that a direct PC-1-PC-2 interaction is required for the complex localization at the cilium [57,58,59]. The model proposed by the Kim *et al.* shows that PC-1 undergoes cleavage at its GPS site before forming a complex with PC-2 in the endoplasmic reticulum. This interaction is necessary for the complex to reach the TGN for subsequent ciliary targeting. Furthermore, they showed that the PC-1/PC-2 complex traffics to cilia through a Rabep1/GGA1/Arl3-dependent mechanism [57]. In line with this finding, PC-2 has been described to act as a chaperone for PC-1 maturation and localization [59]. Furthermore, studying mutant forms of PC-1 and PC-2 *in vitro* and *in vivo* further underlined the importance of the GPS cleavage in PC-1 for proper PCs trafficking to the cilium [58].

Although the primary cilium (a functionally important localization site of PCs), has been identified as the key organelle in the pathogenesis of ADPKD [3], the functional site of the PCs for preventing cystogenesis is still controversial [60]. A large body of evidence from many different laboratories has demonstrated that alterations into the ciliary structure or function, inevitably results in renal cystogenesis. However, studies on the PCs have demonstrated that at least in two distinct cases, lack of the PCs in cilia does not necessarily drive cystogenesis. The group of Dr. Pazour has previously described the Golgi-resident protein GMAP210 which is essential in driving PC-2 trafficking to cilia [61]. Of interest, GMAP210 knockout mice did not show any sign of renal cystogenesis during development and they died perinatally of heart and lung failure [61]. Thus, in this specific case the reduction of PC-2 trafficking to cilia does not correlate with cyst formation during development. However, we cannot exclude the fact that in this specific animal model there is some residual PC-2 at cilia which is below the threshold of detectability, but which is sufficient to prevent cystogenesis. Nevertheless, a second study further supports the idea that perhaps lack of Polycystins at cilia is not necessarily resulting in cystogenesis. The group of Dr. Qian as shown that a mutant PC-1 lacking cleavage at its GPS site does not traffic to cilia [57,62], yet it prevents embryonic renal cyst formation [63], suggesting again that the key role of PC-1 in preventing renal cystogenesis during development is not located in the cilia. Thus, while the central role of cilia in the pathogenesis of most forms of PKD has been proven, it should be considered that at least during embryonic development the ciliary function of the PCs might not be central to prevent cystogenesis.

**Figure 4:**
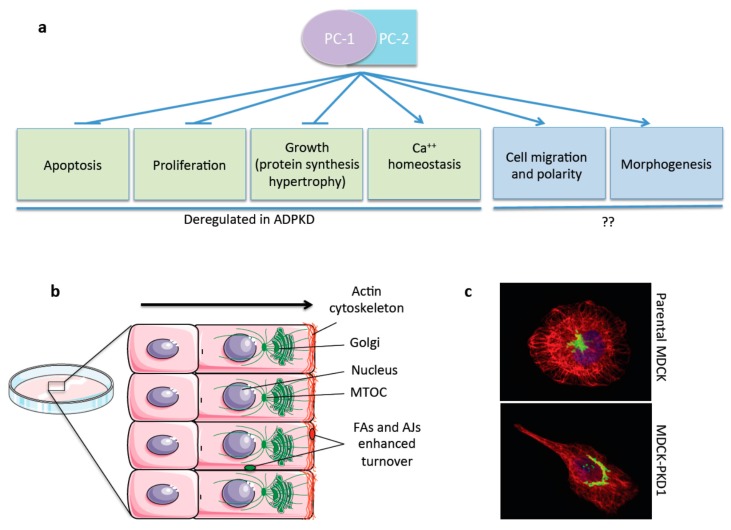
The Polycystins in Cell Migration. PC-1 and PC-2 are involved in different pathways and functional roles, among these they were reported to inhibit apoptosis, proliferation, and growth. Furthermore, they are known to regulate calcium homeostasis. All these processes were shown to be deregulated in ADPKD. Additional functions of the PCs more recently reported involve regulation of cell migration and tissue morphogenesis. The involvement of these last two functions in the disease remains unclear (see text) (**a**); schematic summary showing the cellular effects of the PCs in cell migration and front-rear polarity in wound-healing assays [5,6,8,74]. PC-1 regulates the actin and microtubular cystoskeleton and the turnover rates of FAs and AJs and affecting both the rates of cell migration and front-rear polarity (**b**); at a single cell level MDCKII cells appear asymmetrical, whereas overexpression of PC-1 induces elongation and cellular asymmetry with a polarized actin cytoskeleton (red) and the Golgi (green) re-positioned in front of the nucleus (blue) (**c**).

One major issue that further complicates the understanding of the disease is the fact that the PC-1/PC-2 complex function remains largely unknown, although some progress in recent years has been made. Both PC-1 and PC-2 have been shown to protect cells from apoptosis [64,65] and to inhibit cell proliferation through various pathways [66,67,68] (Figure 4a). It has also been shown that these proteins regulate the protein translation machinery separately: PC-1, acting on S6K1 and S6 ribosomal protein, and the 4EBP1/eIF4E complex [69] and PC-2, acting on eIF2alpha, by regulating its phosphorylation by PERK in response to ER stress [70]. Other several signaling cascades are reported to be controlled by PC-1: the Wnt cascade [71,72], AP-1 [73], PI3kinase/Akt [65,74], GSK3β [74], STAT6 [75], the calcineurin/NFAT [76] pathway, and the ERK and mTOR cascades [67,69]. Recently a role for PC-1 as a cardiomyocyte or smooth muscle cells mechanosensor has been proposed [77,78,79].

One function of the PCs that is persistently been found by several different laboratories and in different cellular and tissue contexts, is their role in regulation of cell migration and tissue morphogenesis [5,6,7,8,64,74,80,81,82] (Figure 4a). We believe this is an essential function of the complex and one that deserves careful attention (see below).

## 5. The Polycystins in Cell Migration, Polarity, and Tissue Morphogenesis

The involvement of PC-1 in regulating cell migration and cellular morphogenesis has been demonstrated by different groups. The over-expression of the *PKD1* cDNA in Madin Darby canine kidney (MDCKtypeII) cells was reported to induce spontaneous tubulogenesis when cells are grown in 3D collagen gels [64]. Subsequently, Nickel *et al.* have shown that the over-expression of the C-terminal tail of PC-1 in murine inner medullary collecting duct (mIMCD3) cells is also able to induce tubulogenesis in 3D cultures and they also showed that the C-tail of PC-1 can regulate cell migration [80]. Subsequent studies have shown that over-expression of PC-1 in MDCK cells results in a strong migratory effect in these cells when challenged by wound-healing [74]. Importantly, PC-1 was found to have a profound effect on the actin cytoskeleton, especially at the leading-edge of migrating cells. The last was reported to depend on the PI3kinase pathway [74]. Finally, these cells showed a more efficient turnover of β-catenin at the adherens junctions, resulting in a reduced mechanical strength of cell–cell adhesion [74] (Figure 4b). In line with some of these studies, subsequent work has suggested that PC-1 regulates the actin cytoskeleton by interacting with Pacsin and WASP-2, stimulating the activation of the complex Arp2/3, involved in the nucleation of microfilaments at the leading edge [7].

Of interest, more recent work has provided evidence that PC-1 regulates cell migration at two distinct levels: not only PC-1 can regulate the rates of cell motility [74,80], but it can also regulate front-rear polarity during cell migration, *i.e.*, the capability of cells to reach a cellular asymmetry and to re-orient the organelles towards the direction of migration [5]. This specific property of PC-1 could be observed both at the single cell level and during collective cell migration in wound healing assays (Figure 4b,c) [5]. In this case, physical interaction with the polarity protein Par3 has been proposed to stand at the basis of regulation of front-rear polarity by PC-1. Importantly, more recent work has confirmed that cells lacking the PC-1 protein have an impaired capability to achieve front-rear polarity during cell migration [6,7]. Notably, PC-1 has been shown to regulate cell motility and front-rear polarity in a variety of cell types including fibroblasts, epithelial cells and endothelial cells [5,6,7,8,83]. Finally, a more recent study has reported that PC-1 regulates the microtubule stability and dynamics in addition to the actin cytoskeleton [8]. The effect of PC-1 on the microtubule cytoskeleton is important for regulation of the turnover rates of focal adhesions and ultimately impact on the cell migratory rates as well as on the adhesion properties of the cells to the substrate [8] (Figure 4b). Interestingly, not the actin cytoskeleton but only the dynamic regulation of the microtubule cytoskeleton seems to be essential in PC-1-mediated cell orientation during migration [8].

The obvious question that arises from the above studies is what could the physiological meaning of this very specific read-out of function that can be observed *in vitro* be? The elegant study by Outeda *et al*. has linked the lack of front-rear polarity in endothelial cells to a remarkably specific phenotype of defective lymphatic vessel morphogenesis both in *Pkd1* and *Pkd2* mutant mice [6]. Similar findings were reported in zebrafish as well [84]. It is well recognized that defective polarized migration in the endothelia of lymphatic vessels results in improper morphogenesis [6,84]. The study by Castelli *et al.* has linked defective oriented cell migration due to improper regulation of the Par3/aPKC complex to a defect in CE movements in the developing kidneys of *Pkd1* mutant mice [5]. Importantly, the authors have shown that a similar defect in CE could be detected in mice carrying UB-specific inactivation of the *Par3* gene, suggesting that at least at the molecular level front-rear polarity and CE share some similarities [5].

Planar cell polarity is a very peculiar type of polarity, which has been very difficult over the years to reproduce *in vitro*. The PCP observed in multi-ciliated cells such as ependymal and bronchial cells is the only one that has so far been recapitulated *in vitro* [85,86]. However, there is some evidence in the literature that front-rear polarity and planar cell polarity share some characteristics, at least at the molecular level, with some core PCP molecules being involved in front-rear polarity [87] and *vice versa*, some front-rear polarity genes being involved in regulation of PCP [88,89]. In this context, one possibility is that the dynamic regulation of the microtubules by PC-1 and the consequent enhanced turnover of both adherens junctions (AJs) and focal adhesions (FAs) might be relevant in the context of CE during tissue morphogenesis when cell-cell junctions need to be remodeled to achieve proper cell intercalation (Figure 3 and Figure 4b).

Aside from its role in regulation of CE and, presumably, PCP in the developing renal tubule, what is the function of the PCs complex in more classical examples of PCP? Inactivation of the *Pkd1* gene in the cochlea resulted in defective morphology of the stereocilia, but no defect in the planar polarity properties of the epithelial cells in the sertoli organ [90]. This finding has been viewed as evidence that PC-1 does not regulate PCP. However, a very elegant study has more recently demonstrated that PC-1 can regulate planar polarity in ependymal cells [91], although it remains unclear whether this can be considered a direct effect that PC-1 plays in a cell autonomous manner in the ependymal epithelia or whether this might be the result of a more indirect regulation [91].

## 6. Defective Planar Cell Polarity as a Cause of Cystogenesis?

Importantly, defective PCP has been proposed to stand at the basis of CKD [92]. One of the first indications of a link between CKD and PCP can be found in a recessive cystic kidney disease known as nephronophthisis (NPHP) [2], in which inversin, one of the genes mutated, controls the balance between canonical and non-canonical Wnt signaling in response to cilia bending and is related to the core PCP protein Dgo [93]. Subsequently, Fischer *et al.* reported the first experimental evidence of defective PCP in PKD, examining two different rodent models of PKD and finding a loss of OCD in mutants [41]. They proposed that defects in mitotic spindle orientations found in the pck rats (a model of Autosomal Recessive Polycystic Kidney Disease, ARPKD) lead to a failure in the regulation of tubular diameter causing dilatation and cyst formation [41]. These observations demonstrated for the first time the presence of OCD in the renal tubules and its essential role in maintenance of a proper tubular diameter in the rodent renal tubules [41]. Several studies have subsequently confirmed the presence of OCD in wild-type tubules and its essential role in proper tubular elongation [94,95]. Saburi *et al.* observed that loss of Fat4 disrupts PCP signaling and OCD leading to CKD [95]. In addition to all of these studies, Karner *et al.* observed that attenuation of Wnt9b signaling in mice during kidney morphogenesis affects PCP of the epithelium leading to tubules characterized by a larger diameter due to defective CE, which eventually cause defective cell intercalation and consequent cyst formation [42]. Finally, live-imaging of the PCP-dependent elongation of renal tubules in the developing nephron in *Xenopus in vivo*, showed that disrupted rosette topology is accompanied by a reduction in nephron elongation and defective tubular diameter [44]. All these studies taken together seem to suggest that defective PCP and cyst formation appear to be tightly linked.

However, while the central role of PCP in the developing renal tubule has been unequivocally proven, its role in cystogenesis is less clear. The fact that inactivation of the central PCP molecule Fat4 in the kidney leads to cyst formation is perhaps the strongest evidence generated to date to suggest that disruption of PCP in the kidney leads to cyst formation [95]. Further to this, the fact that disruption of key molecules causing cystogenesis in the mouse led to defective PCP also points to a possible central role of this biological process in cyst formation. Mutations in HNF1beta showed a correlation between misoriented cell division and cyst formation [41]. Likewise, Luyten *et al.* showed that mice lacking *Pkd1* in their distal tubule segments also have defects in OCD in kidney tubules [96]. However, several evidences have also been provided that question the centrality of PCP in cystogenesis: (i) first, a second study on *Pkd1* mutants failed to identify randomization of the mitotic spindles in the pre-cystic kidneys and only reported mis-orientation in the already cystic lesions [94]; similar results of cyst formation in the absence of misoriented cell division in pre-cystic tubules was also observed in *ift140* mutant mice [97]; (ii) second, a hypomorphic allele for a core PCP molecule such as Vangl2 in the kidney disrupts correct morphogenesis and causes mild tubular dilatations, but does not result in an overt cystic phenotype [98]. This is not, *per se*, a strong evidence against the model as one cannot exclude the possibility that in higher organisms the molecular determinants of PCP are more diversified than they are in lower organisms. Nevertheless these studies should be considered when thinking of the problem; (iii) third, an elegant study by Nishio *et al.* has taken advantage of a mouse model carrying mutations in the *Pkhd1* gene, responsible for ARPKD [94]. These mice lack entirely the function of the polyductin/fibrocystin protein, however for unclear reasons they fail to develop renal cystogenesis. This mouse model showed defective OCD [94]. The data demonstrate that yet another gene mutated in cystic kidney disorders can regulate PCP in the developing nephron. However, they also show that impairment of this property of renal epithelia is not sufficient to drive cystogenesis. The authors have shown that in this specific case compensatory mechanisms such as cellular extrusion followed by intercalation can compensate for defective OCD and help maintain a proper tubular diameter; (iv) fourth, a second report has concentrated on studying the role of the *Pkd1* gene in the process of CE during renal development [5]. A defect in the achievement of proper tubular diameter was identified likely due to defective CE and cell intercalation [5]. In this case, cystogenesis was observed. However, in the same study the authors show that a key interactor of PC-1, *i.e.*, Par3, when inactivated in the kidney, shows a very profound defect in tubular narrowing and CE in 100% of the animals analyzed, while cystogenesis could be observed only in a minority of renal samples (40%) [5]. These data further strengthen the evidence that cystic disease genes can regulate PCP processes, but again, they also show that dysregulation of PCP-dependent processes in the developing kidney seems not to be sufficient to drive cystogenesis. In this case, as well, the authors hypothesize that a compensatory mechanism might be involved.

All together, these data show that regulation of PCP appears to be central in the function of cystic disease genes, but alteration of one PCP process at a time might not be sufficient to drive cystogenesis. It would be extremely interesting to test whether combining mutants that are, *per se*, minimally cystic but show defective CE (Par3) with those mutants that are non-cystic but show defective OCD (*Pkhd1*) is sufficient to recapitulate highly-penetrant cystogenesis in the mouse.

## 7. Concluding Remarks

A large body of evidence was accumulated that suggests that the PCs play a key role in directing cell migration and polarity, which is most likely essential to achieve proper morphogenesis in several tissues, including the lymphatic vessels and the renal tubules. Additionally, increasing evidence suggests that planar polarity might be influenced by the PCs and by all cystic disease genes, in a process essential for proper tubular morphogenesis during development. However, while it has been proposed that the cysts in ADPKD can develop during the embryonic life of a kidney [99], it is also likely that loss of function of the PKD genes in the adult tubule is sufficient to drive cystogenesis. Thus, what could be the role of regulation of PCP in the context of a mature tubule? It has been proposed that tissue damage can be a driver of cystogenesis and act as an additional insult (providing a so-called “third hit” in ADPKD [100]) able to accelerate or induce cystogenesis. In the mouse, ischemic injury both accelerates cystogenesis and also causes a defective cellular orientation and cell polarity [101]. Thus, one possibility is that all these programs of morphogenesis that are essential during development are re-activated in response to an insult and are essential for proper tissue repair. These insults might be of very different origin in addition to ischemic injury, including environmental factors, aging, and stress that could cause the need for tissue regeneration. The function of the PCs in this context might be to maintain an appropriate plasticity in the mature epithelia (or endothelia), which might be helpful during tissue repair.

One additional interesting aspect that is emerging from these recent studies is the possible involvement of cilia in driving PCP and even oriented and collective cell migration. Further studies would be important to understand what the role of cilia during renal tubular development is or whether their function during tubular morphogenesis is, instead, dispensable.

However, the most important aspect that deserves careful attention from the scientific community is whether the role of the PCs in regulation of cellular shape and polarity is indeed essential for preventing renal cystogenesis or not. This would have important implications for therapy as well. It is possible that this specific function of the complex is not so essential for preventing cystogenesis. If it is the case, however, and the primary defect underlying cystogenesis is a geometrical and structural defect of the epithelia, then this might be a very difficult problem to correct by employing a pharmacological approach. One could, in principle, think of restoring proper expression and or/trafficking of PC-1 when the mutations are mild, but restoring the truncated protein (present in approximately 40% of patients) will not be feasible [102]. Thus, from a therapeutic point of view for all the more severe mutations we will be left with the need to target secondary dysfunctions of the cystic epithelia.
